# High-Flow Nasal Oxygen versus Noninvasive Ventilation in Acute Exacerbation of Chronic Obstructive Pulmonary Disease Patients: A Meta-Analysis of Randomized Controlled Trials

**DOI:** 10.1155/2023/7707010

**Published:** 2023-06-30

**Authors:** Yanping Du, Huaping Zhang, Zhiyi Ma, Jun Liu, Zhiyong Wang, Meixia Lin, Fayu Ni, Xi Li, Hui Tan, Shifan Tan, Yanling Chai, Xiangzhu Zhong

**Affiliations:** ^1^The School of Clinical Medicine, Fujian Medical University, Zhongshan Hospital Xiamen University, Fujian, China; ^2^Pulmonary and Critical Care Medicine, Second Affiliated Hospital of Fujian Medical University, Respiratory Medicine Center of Fujian Province, Fujian, China; ^3^Pulmonary and Critical Care Medicine, The First Hospital of Longyan Affiliated to Fujian Medical University, The School of Clinical Medicine, Fujian Medical University, Fujian, China; ^4^Pulmonary and Critical Care Medicine, The School of Clinical Medicine, Fujian Medical University, The Second Hospital of Longyan, Fujian, China; ^5^Pulmonary and Critical Care Medicine, The School of Clinical Medicine, Fujian Medical University, The First Hospital of Putian, Fujian, China; ^6^Pulmonary and Critical Care Medicine, Fuqing Hospital Affiliated to Fujian Medical University, Fujian, China; ^7^Pulmonary and Critical Care Medicine, The Second People's Hospital Affiliated to Fujian Traditional Chinese Medicine, Fujian, China; ^8^Pulmonary and Critical Care Medicine, Chenzhou No. 1 People's Hospital, Hunan, China; ^9^Pulmonary and Critical Care Medicine, Maoming People's Hospital, Guangdong, China; ^10^Pulmonary and Critical Care Medicine, The Second Affiliated Hospital of Kunming Medical University, Yunnan, China; ^11^Pulmonary and Critical Care Medicine, Foshan Fosun Chancheng Hospital, Guangdong, China

## Abstract

**Background:**

High-flow nasal cannula (HFNC) can be used in stable chronic obstructive pulmonary disease (COPD) patients, but the effect of HFNC on clinical outcomes in patients with acute exacerbation of chronic obstructive pulmonary disease (AECOPD) is still uncertain.

**Methods:**

We searched electronic literature databases for randomized controlled trials (RCTs) comparing HFNC with noninvasive ventilation (NIV) in hypercapnic patients with AECOPD. The primary endpoint of this meta-analysis was PaCO_2_, PaO_2,_ and SpO_2_. The secondary outcomes were the respiratory rate, mortality, complications, and intubation rate.

**Results:**

We included 7 RCTs with a total of 481 patients. There were no significant differences on measures of PaCO_2_ (MD = −0.42, 95%CI −3.60 to 2.75, *Z* = 0.26, and *P* = 0.79), PaO_2_ (MD = −1.36, 95%CI −4.69 to 1.97, *Z* = 0.80, and *P* = 0.42), and SpO_2_ (MD = −0.78, 95%CI −1.67 to 0.11, *Z* = 1.72, *P* = 0.08) between the HFNC group and the NIV group. There was no significant difference in measures of the mortality and intubation rate between the HFNC group (OR = 0.72, 95%CI 0.30 to 1.69, *Z* = 0.76, and *P* = 0.44) and the NIV group (OR = 2.38, 95%CI 0.49 to 11.50, *Z* = 1.08, and *P* = 0.28), respectively. But the respiratory rate in the HFNC group was lower than that in the NIV group (MD = −1.13, 95%CI −2.13 to −0.14, *Z* = 2.23, and *P* = 0.03), and fewer complications were found in the HFNC group (OR = 0.26, 95%CI 0.14 to 0.47, *Z* = 4.46, and *P* < 0.00001).

**Conclusion:**

NIV was noninferior to HFNC in decreasing PaCO_2_ and increasing PaO_2_ and SpO_2_. Similarly, the mortality and intubation rate was similar among the two groups. The respiratory rate and complications were inferior in the AECOPD group treated with HFNC.

## 1. Introduction

The high-flow nasal cannula (HFNC) is a high-concentration oxygen technique that reliably achieves a FiO_2_ as high as 100%. HFNC is more likely to benefit patients with severe symptoms who need high-concentration oxygen rather than patients requiring low oxygen flow rates [1]. So, HFNC is more frequently used in severe acute respiratory failure patients who are at high risk of intubation. But for hypercapnic respiratory failure secondary to acute exacerbation of chronic obstructive pulmonary disease (AECOPD), the standard of care is noninvasive ventilation (NIV) [2]. In some patients, NIV is sometimes difficult to tolerate. Especially younger patients with breathing frequency and a high heart rate may more frequently experience NIV intolerance [3]. There is a need for providing another treatment option that is both easy to administer and well tolerated for hypercapnic chronic obstructive pulmonary disease (COPD) patients. The benefits of HFNC include providing oxygen at high flows with an optimal degree of heat, carbon dioxide washout of upper airways by providing airflows 30−60 L/min, and enhanced secretion clearance through the provision of reliable humidification [4]. In one investigation with COPD patients treated for home HFNC in a crossover clinical trial, HFNC with the titration of 20 L/min was not inferior to NIV in COPD patients with stable hypercapnia for a lower CO_2_ clearance [5]. A meta-analysis in stable hypercapnic COPD patients comparing HFNC to conventional oxygen demonstrated that the addition of HFNC did not increase the arterial partial pressure of carbon dioxide (PaCO_2_) in these patients [6]. However, the effect of HFNC on clinical outcomes in patients with AECOPD with hypercapnia is still uncertain. We performed a meta-analysis to compare the clinical outcomes for HFNC with NIV in the AECOPD patients.

## 2. Methods

### 2.1. Search Strategies

We search electronic literature databases for randomized controlled trials (RCTs) comparing HFNC with NIV in hypercapnic patients with AECOPD. A literature search was performed through the following databases: Cochrane, Google Scholar, MEDLINE database, PubMed, and Embase from inception to July 2022. The following search terms were used: high-flow nasal cannula or nasal high-flow therapy or high-flow nasal cannula or high-flow oxygen through nasal cannula or high-flow nasal therapy, non-invasive ventilation or noninvasive ventilation or noninvasive positive pressure ventilation, acute exacerbation of obstructive pulmonary disease or COPD patients with chronic respiratory failure or hypercapnic respiratory failure or chronic obstructive pulmonary disease patients with hypercapnia or COPD exacerbation, and randomized controlled trial or randomized clinical trial.

### 2.2. Data Extraction

Two reviewers independently evaluated the included studies and extracted data into RevMan 5.3 (review manager: Cochran handbook for systematic reviews). Any disagreement about whether the RCT met the inclusion or exclusion criteria between the two reviewers was resolved by discussing it with a third reviewer. If still more information was required, communication with the authors through Email would be carried out.

### 2.3. Study Selection

We included RCTs comparing HFNC with NIV in AECOPD patients. The inclusion criteria included (1) randomized control trials, (2) human studies, (3) the comparison between HFNC with NIV in hypercapnic patients with AECOPD was performed in the study, (4) all patients were adults, and (5) if more than one eligible study using the same protocol from the same centre, the study with the longest follow-up was used. The exclusion criteria were as follows: (1) studies reported none of these outcomes: PaCO_2_, PaO_2_, SpO_2_, respiratory rate, mortality, complications, and intubation rate. (2) We excluded studies with the baseline pH < 7.30, for most of these patients need invasive ventilation.

### 2.4. Outcome Measure

The primary endpoint of this meta-analysis was PaCO_2_, PaO_2_, and SpO_2_. The secondary outcomes were the respiratory rate, mortality, complications, and intubation rate.

### 2.5. Quality Assessment

We used the Cochran handbook for systematic reviews of intervention guidelines to assess the risk of bias. Each study was evaluated for random sequence generation, concealment of allocation sequence, blinding of the participants and personnel, blinding of outcome assessment, incomplete outcome, and selective reporting. Also, they were classified by two authors as having a high risk of bias and unclear risk of bias or a low risk of bias based on the Cochrane tool.

### 2.6. Statistical Analysis

Statistical analysis of the meta-analysis was done using Cochrane systemic review software RevMan 5.3. We used the Mann–Whitney *U* test to help us verify the hypothesis and rendered statistical significance as a *P* value and a *Z* value < 0.05. The odds ratio (OR) and 95% confidence intervals (CIs) were calculated for dichotomous outcomes, and weighted mean differences (WMDs) and 95% confidence intervals (CI) were calculated for continuous outcomes in each included study. The *I*^2^ value was used to assess statistical heterogeneity. If the *I*^2^ value ≤ 50% was considered as having no statistical heterogeneity, a fixed effects model was used to estimate the overall summary effect sizes. Otherwise, we used a random effects model. And subgroup analysis or sensitivity analysis would be carried out.

## 3. Results

### 3.1. Study Selection

**Figure 1:**
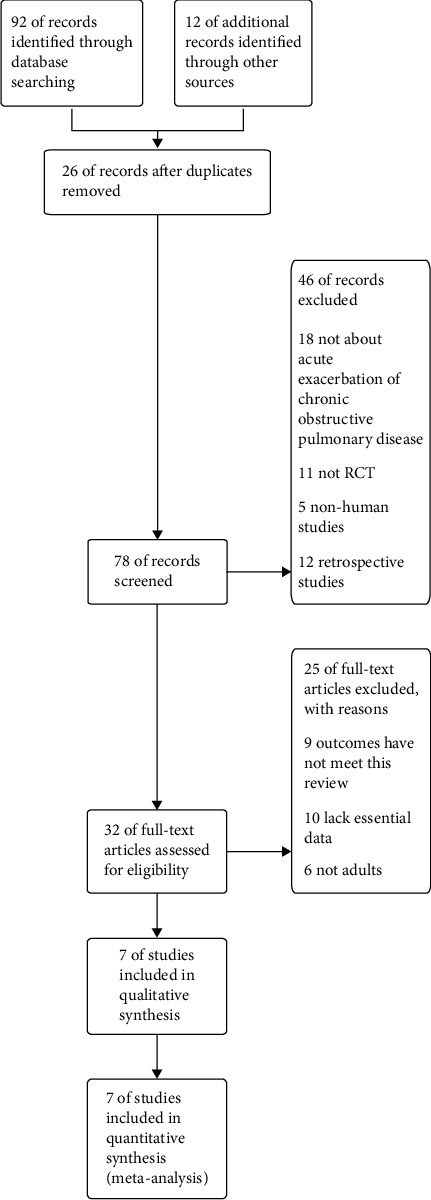
Flow diagram of the details search and exclusion criteria.

The search algorithm identified 104 records. 92 records were identified from electronic databases and 12 records from reference lists. After deduplication, 26 records were excluded. 78 records were screened. 46 records were excluded by reading the abstracts for not about acute exacerbation of COPD (*n* = 18), not RCT (*n* = 11), nonhuman studies (*n* = 5), and retrospective studies (*n* = 12). 32 full-text articles were assessed for eligibility. 25 full-text articles were excluded for reasons. 9 articles were excluded for outcomes that have not met this review, 10 articles were excluded for lack of essential data, and 6 articles were excluded for not adults. 7 articles were included in the final meta-analysis [7–13] Figure 1.

### 3.2. Included Studies

We included 7 RCTs with a total of 481 patients. All included studies had been published and is shown in Table 1.

### 3.3. Quality Assessment

**Figure 2:**
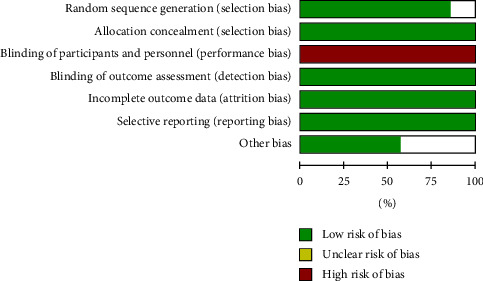
The risk of the bias graph.

**Figure 3:**
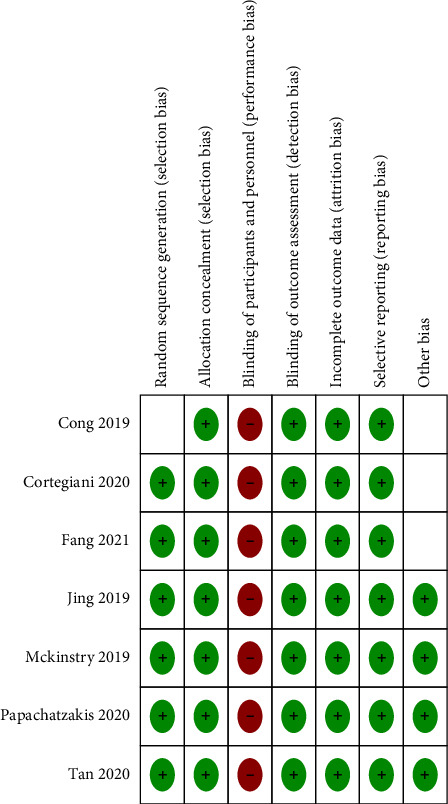
The risk of the bias summary.

The risk of bias about the methodological quality of the included studies are elaborated and summarized, respectively, in Figures 2 and 3. Due to the nature of respiratory support management, blinding the participants is not possible.

### 3.4. Heterogeneity

No statistical heterogeneity was found between the HFNC and NIV groups in PaO_2_ (*I*^*2*^ = 0%, chi = 1.21, and *P* = 0.55), SpO_2_ (*I*^*2*^ = 18%, chi = 2.44, and *P* = 0.30), respiratory rate (*I*^*2*^ = 10%, chi = 4.45, and *P* = 0.35), mortality (*I*^*2*^ = 0%, chi = 1.04, and *P* = 0.79), and complications (*I*^*2*^ = 0%, chi = 1.90, and *P* = 0.39); so, a fix effects model had been used. Statistical heterogeneity was found between the HFNC and NIV groups in PaCO_2_ (*I*^*2*^ = 56% chi = 9.13, and *P* = 0.06) and the intubation rate (*I*^*2*^ = 51% chi = 4.12, and *P* = 0.13); so, a random effects model had been used.

### 3.5. Effect of the Intervention

#### 3.5.1. The Primary Endpoint

**Figure 4:**
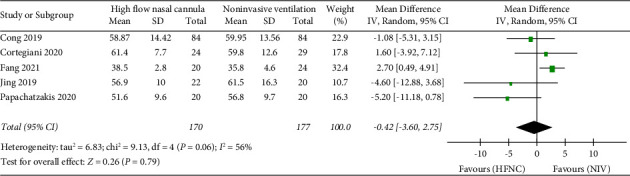
Forest plot of standardized mean difference with a confidence interval for PaCO_2_.

*(1) PaCO_2_*. The primary endpoint is PaCO_2_, PaO_2,_ and SpO_2_. “PaCO_2_” was reported in five studies. 170 patients in the HFNC group and 177 patients in the NIV group were available to compare the PaCO_2_. There was no significant difference in measures of PaCO_2_ between the HFNC group and the NIV group (MD = −0.42, 95%CI −3.60 to 2.75, *Z* = 0.26, and *P* = 0.79), Figure 4.

**Figure 5:**
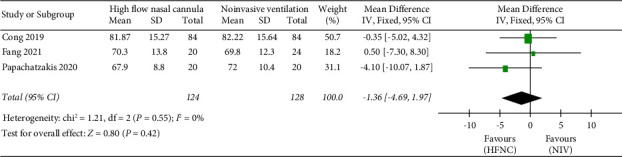
Forest plot of standardized mean difference with a confidence interval for PaO_2_.

*(2) PaO_*2*_*. The “PaO_2_” was reported in three studies. 124 patients in the HFNC group and 128 patients in the NIV group were available to compare the PaO_2_. There was no significant difference in measures of PaO_2_ between the HFNC group and the NIV group (MD = −1.36, 95%CI −4.69 to 1.97, *Z* = 0.80, and *P* = 0.42), Figure 5.

**Figure 6:**
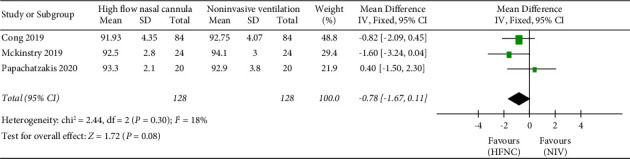
Forest plot of standardized mean difference with a confidence interval for SpO_2_.

*(3) SpO_*2*_*. The “ SpO_2_” was reported in three studies. 128 patients in the HFNC group and 128 patients in the NIV group were available to compare the PaO_2_. There was no significant difference in measures of SpO_2_ between the HFNC group and the NIV group (MD = −0.78, 95%CI −1.67 to 0.11, *Z* = 1.72, and *P* = 0.08), Figure 6.

#### 3.5.2. The Secondary Endpoint

**Figure 7:**
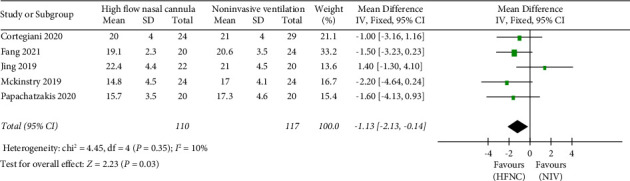
Forest plot of standardized mean difference with a confidence interval for the respiratory rate.

*(1) Respiratory rate*. The second endpoint contains three outcomes: respiratory rate, mortality, and complications. First, the respiratory rate was reported in four studies. 110 patients in the HFNC group and 117 patients in the NIV group were available to compare the respiratory rate. The respiratory rate in the HFNC group was lower than that in the NIV group (MD = −1.13, 95%CI −2.13 to −0.14, *Z* = 2.23, and *P* = 0.03), Figure 7.

**Figure 8:**
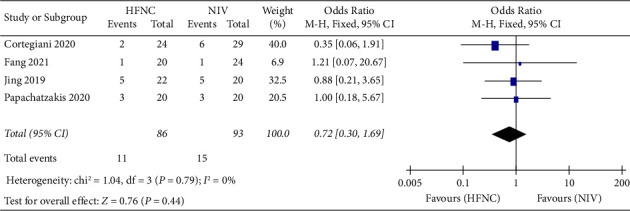
The graph shows a forest plot of the relative risk with a confidence interval for the mortality.

*(2) Mortality*. Second, Mortality was reported in three studies. 86 patients in the HFNC group and 93 patients in the NIV group were available to compare the respiratory rate. There was no significant difference in measures of mortality between the HFNC group and NIV group (OR = 0.72, 95%CI 0.30 to 1.69, *Z* = 0.76, and *P* = 0.44), Figure 8.

**Figure 9:**
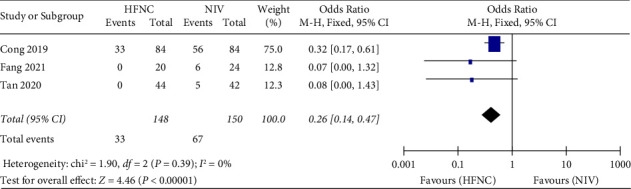
Forest plot of the relative risk with a confidence interval for the complications.

*(3) Complications*. Third, complications were reported in three studies. 148 patients in the HFNC group and 150 patients in the NIV group were available to compare the respiratory rate. Fewer complications were found in the NIV group (OR = 0.26, 95%CI 0.14 to 0.47, *Z* = 4.46, and *P* < 0.00001), Figure 9.

**Figure 10:**
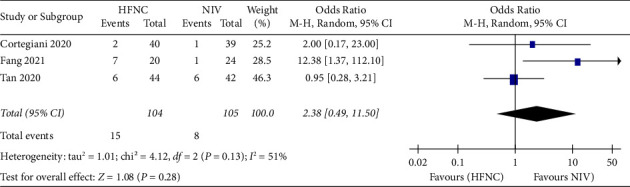
Forest plot of the relative risk with a confidence interval for the intubation rates.

*(4) Intubation rates*. Fourth, intubation rates were reported in three studies. 104 patients in the HFNC group and 105 patients in the NIV group were available to compare the respiratory rate. Fewer complications were found in the NIV group (OR = 2.38, 95%CI 0.49 to 11.50, *Z* = 1.08, and *P* = 0.28), Figure 10.

## 4. Discussion

The major finding in our meta-analysis was that HFNC is not inferior to NIV (which is the standard of treatment for acute decompensated hypercapnic respiratory failure) in decreasing PaCO_2_ and increasing PaO_2_ and SpO_2_. Similarly, the mortality and intubation rate was similar among the two groups. The respiratory rate and complications were inferior in the AECOPD group treated with HFNC.

NIV is the standard treatment for hypercapnic respiratory acidosis patients according to current guidelines and can significantly reduce mortality and the need for intubation among AECOPD patients with respiratory acidosis. This benefit appears similar for patients with a mild (pH 7.30 to 7.35) and a more severe nature (pH < 7.30) [14]. NIV corrects the mechanism leading to hypercapnia by increasing the tidal volume and by decreasing the work of breathing while reducing CO_2_ [15]. Many studies indicated that the early use of NIV in mild AECOPD patients with a partial pressure of carbon dioxide (PaCO_2_) >45 mmHg and a pH > 7.25 can effectively alleviate respiratory muscle fatigue. However, intolerability and discomfort have restricted the widespread application of NIV in AECOPD patients [16].

HFNC oxygen therapy is used in hypoxemic respiratory failure patients. But some studies have explored that different flow rates of HFNC resulted in a flow-dependent reduction in PaCO_2_ in stable hypercapnic COPD patients [17]. For safety and efficacy, HFNC has not been demonstrated in AECOPD patients. When compared to NIV, HFNC also demonstrated a reduction of the inspiratory muscle effort similar to spontaneous breathing [18]. Recent studies found that the short-term (within 2 h) application of HFNC could effectively decrease PaCO_2_. Several mechanisms are involved in the explanation of these results, such as the reduction of the anatomical dead space in the upper airways and inspiratory resistance, which improves alveolar ventilation [19]. The adequate flow and humidified warm gas can attenuate inspiratory resistance and increase expiratory resistance can also reduce the physiological dead space, improve airway clearance, and attenuate the work of breathing, allowing for a higher fraction of minute ventilation to facilitate gas exchange [1]. At the same time, HFNC also increases the tidal volume to a lesser extent than NIV. HFNC improves the washout of the upper airway dead space and generates a low level of positive end-expiratory pressure (PEEP) [20]. Other studies found HFNC led to a flow-dependent reduction in PaCO_2_, accompanied by an increase in the tidal volume [21]. A recent study found HFNC combined with NIV had a higher degree of comfort in patients with AECOPD, can improve blood gas parameters, and increase patient compliance [22].

HFNC may be better tolerated than NIV. A recent study found blood pressure significantly decreased after using NIV for NIV had more impact on venous return than HFNC [9]. In our meta-analysis, we found that the respiratory rate in the HFNC group was lower than that in the NIV group. Other studies have explored the physiological effects of HFNC, and HFNC could decrease the neuroventilatory drive and work of breathing in COPD patients [23]. This can explain why the respiratory rate in the HFNC group was lower, and the patients felt more comfortable than the NIV group. We also found that fewer complications were found in the HFNC group. A retrospective study found that there were fewer nursing interventions and skin breakdown episodes reported in the HFNC group compare to the NIV group [24]. Some studies found that more patients needed bronchoscopy for secretion management in the NIV group since the patients on NIV feel uncomfortable and claustrophobic within the secured mask. This may be due to the inconvenience of coughing by taking the mask off. By contrast, the humidifying and heating function of HFNC enables the gas delivered to reach a temperature of 37°C and an absolute humidity of 44 mg H_2_O/L, and HFNC can provide optimal humidity, so patients can drink, cough, and talk [25]. For this easy compliance, a subgroup analysis found fewer patients required intubation in the HFNC group than in the NIV group, although this was not a statistically significant finding [26]. But in our meta-analysis, we found complications that were statistically fewer in the HFNC group than in the NIV group.

## 5. Limitations

This study had two limitations. First, most studies used AIRVO™ 2, Fisher and Paykel Healthcare, Auckland, New Zealand device except the Cong 2019 study. Also, the NIV device included Philips Respironics BiPAP AVAPS-ST 60 Series, ResMed, Bella Vista, NSW, Australia, and other devices. These different devices can cause bias. Second, due to the nature of respiratory support management, blinding the participants is not possible.

## 6. Conclusion

NIV was noninferior to HFNC in decreasing PaCO_2_ and increasing PaO_2_ and SpO_2_. Similarly, the mortality and intubation rate was similar among the two groups. The respiratory rate and complications were inferior in the AECOPD group treated with HFNC.

## Figures and Tables

**Table 1:** Characteristics of the seven randomized controlled trials included in the meta-analysis.

References	Study designs	Countries	Total (*n*) HFNC/NIV	Participants	HFNC device	NIV device	Age (HFNC/NIV)	Setting of HFNC and NIV	Duration of HFNC or NIV	Outcomes
Mckinstry et al. 2019 [8]	Single-center crossover RCT	New Zealand	24	Eligible participants were aged at least 40 years, had a doctor's diagnosis of COPD and had a capillary partial pressure of carbon dioxide of ˃45 mmHg	AIRVO 2 device (PT101AZ, fisher and paykel healthcare, Auckland, New Zealand)	Philips respironics BiPAP AVAPS-ST 60 series (Philips Respironics, Eindhoven, Netherlands)	68 ± 9.0	All participants received an AB/BA crossover design. HFNC was delivered at a flow rate of 45 L/min and a temperature of 37°C. NIV settings were delivered at an inspiratory pressure of 15 cm H_2_O and expiratory pressure of 4 cm H_2_O	Participants received both HFNC and NIV for 60 min each, while seated, followed by a 15 min to allow the PtCO_2_ to return to within 2 mm Hg of the baseline measurement	The transcutaneous partial pressure of carbon dioxide (PtCO_2_), respiratory rate, oxygen saturation, heart rate, borg dyspnea scores, tolerability questionnaires
Jing et al. 2019 [9]	Single-centre RCT	China	22/20	COPD patients who were intubated for exacerbation, with hypercapnia (PaCO_2_ ˃45 mmHg) at the time of extubation and met the pulmonary infection control window” criteria were recruited	Optiflow or AIRVO^TM^ 2 device (PT101AZ, fisher and paykel healthcare, Auckland, New Zealand)	Noninvasive ventilator (VPAP III ST; ResMed, Bella Vista, NSW, Australia	77.4 ± 6.8/73.9 ± 6.9	HFNC was utilized with the humidifier temperature set at 37°C and the fraction of inspired oxygen adjusted to maintain oxygen saturation recorded by pulse oximetry (SpO_2_) at 88–92%. For NIV, its standard oral-nasal mask was used. Inspiratory positive airway pressure was initiated at 10−12 cm H_2_O, and subsequent adjustments were based on the patients' ABGs. Oxygen was blended via the port on the mask and adjusted to maintain SpO_2_ 88–92%	Both HFNC and NIV were used at least 8 hr/day	The primary outcome parameters were ABGs (PH, PaCO_2_, partial pressure of oxygen in arterial blood/fraction of inspired oxygen (PaCO_2_/FiO_2_) and vital signs (heart rate), mean arterial pressure (MAP), respiratory rate (RR) at 3, 24, and 48 hr after extubation. Secondary outcomes included the duration of respiratory support, length of ICU stay, the patient's comfort score, the incidence of adverse events, and the number of patients who needed bronchoscopy for secretion removal within 48 hr after extubation
Papachatzakis et al. 2020 [10]	Single-centre RCT	Greece	20/20	We enrolled patients who were admitted to the hospital because of acute respiratory failure type 2 (PaCO_2_ ˃45 mmHg)	HFNC: optiflow nasal interface connected to the PT101AZ (Airvo2) humidifier fisher and paykel healthcare, Auckland, New Zealand)	NIV: synchrony, respironics, inc, Murrysville, Pennsylvania	76.0 ± 13.4/78.1 ± 8.1	HFNC therapy typically was initiated at a flow of 35 L/min, titrating flow upward if tolerated to 45−50 L/min, to maintain SaO_2_ > 90% or according to specific clinical orders. NIV was initiated by bilevel positive airway pressure (BiPAP) supplied with an identical set of modes. Expiratory and inspiratory pressures were gradually increased to the maximum tolerated over 1 h, to maintain Sao_2_ > 90%, or according to specific clinical orders	Both HFNC and NIV were used during hospitalization (days) 11.5 ± 8.5	We measured and recorded vital signs, arterial blood gases (ABGs), and comfort. Endpoints to evaluate after HFNC or NIV therapy were intubation and mortality rate, length of hospitalization, duration of therapy, and possible differences between vital signs, ABGs, and comfort
Cortegiani et al. 2020 [11]	Multicentre RCT	Italy	24/29	Patients with a diagnosis of COPD exacerbation according to GOLD criteria were admitted for mild-to-moderate acute hypercapnic respiratory failure, with an arterial PH between 7.25 and 7.35 and a PaCO_2_ ≥ 55 mmHg	Optiflow and MR850 or AIRVO^TM^ 2, Fisher and Paykel Healthcare, Auckland, New Zealand		74 ± 13/77 ± 12	HFNC: initially set at 60 L/min and at a temperature of 37°C. In case of discomfort, flow and/or temperature were down-regulated to the most tolerated setting. NIV: the ventilation was set in pressure support ventilation (PSV) mode, with a PEEP titrated between 3 and 5 cm H_2_O. The inspiratory pressure was titrated to achieve a measured or estimated expiratory tidal volume equal to 6–8 mL kg^−1^ of the ideal body weight	All patients received the assigned treatment after randomization. By 2 h, six patients in the HFNC group had switched to NIV and one to IMV in the HFNC group. Due to the improvement of respiratory failure, one patient switched to no support in the HFNC group while two patients switched to HFNC, and six to no support in the NIV group	The primary endpoint was the mean difference of PaCO_2_. (2) Treatment change rates. (3) Dyspnea score and proportion of patients who did not improve the dyspnea score. (4) Discomfort score and proportion of patients showing poor tolerance to treatment. (5) The proportion of patients who had PaCO_2_ worsening or reduction <10 mmHg from baseline assessment, or worsening or no improvement of the dyspnea. (6) Respiratory rate. (7) Change in arterial blood gases. (8) Time spent under mechanical ventilation (both IMV and NIV). (9) Hospital length of stay. (10) Hospital mortality
Tan et al. 2020 [12]	Multicentre RCT	China	44/42	COPD patients with hypercapnic respiratory failure who received invasive ventilation were screened for enrollment. Other inclusion criteria included patients who were ≤85 years of age, able to care for themselves, and met the criteria of the pulmonary infection control window	AIRVO^TM^ 2, Fisher and Paykel Healthcare, Auckland, New Zealand	Philips V60 or BiPap vision	68.4 ± 9.3/71.4 ± 7.8	HFNC: the initial airflow was set at 50 L/min and adjusted according to patient tolerance. The HFNC was set to an absolute humidity of 44 mg H_2_O/L, the temperature was set to 37°C, and FiO_2_ was adjusted to maintain a SpO_2_ of 88–92%. NIV: all subjects receiving NIV were set in S/T mode with a standard oral-nasal (full-face) mask (RT040). The initial expiratory pressure airway pressure was set to 4 cm H_2_O, and the pressure level was gradually increased to ensure that the patient could trigger the NIV device with each inhalation. The inspiratory airway pressure was initially set to 8 cm H_2_O and gradually increased to achieve a satisfactory tidal volume with acceptable tolerance	The patient's initial respiratory support was targeted to last at least 2 h and then continued as needed. NIV or HFNC were discontinued when the total daily treatment duration was less than 4 h and could be reused if needed. Treatment success was defined as no need for respiratory support within 72 h after stopping NIV or HFNC	The primary outcome was treatment failure. Secondary outcomes included arterial blood gas analysis and vital signs such as the respiratory rate, heart rate, and blood pressure, the daily number of nursing airway care interventions, the patient's comfort score, the patient dyspnea score, the incidence of nasofacial skin breakdown, 28-day mortality, and total ICU and hospital lengths of stay
Cong et al. 2019 [7]	Single-centre RCT	China	84/84	The patients were aged 40 to 76 years and the duration of COPD was 2 to 18 years. Patients were included if they were diagnosed with AECOPD according to the national guideline. Admitted to ICU due to severe illness and given ventilation therapy	Patients were ventilated using OH–60°C high-flow noninvasive breathing therapeutic apparatus (Micomme, Hunan, China)	Patients were ventilated by mouth and nose using a ventilator Hamilton G5 (Hamilton medical AG; Bonaduz, Switzerland	The patients were aged 40 to 76 years with an average of 67.51 ± 7.13 years	HFNC: the air temperature was set at 37°C at a flow rate of 30–35 L/min. For NIV, the inspiratory positive airway pressure (IPPV) was set at 10 cm H_2_O, and expiration pressure was set at 5 cm H_2_O at the beginning, and gradually increased after the patient adapted	Both HFNC and NIV were used during hospitalization	The primary endpoint was blood gases as a measure of systemic inflammation during AECOPD and was measured before, 12 h, and 5 days after treatments. Secondary clinical endpoints included ventilation support time, hospitalization days and complications, comfort, and nursing satisfaction. Comfort and nursing satisfaction were investigated using hospital-designed questionnaires
Fang et al. 2021 [13]	Single-centre RCT	China	20/24	Patients with a diagnosis of COPD exacerbation according to GOLD criteria were admitted for mild-to-moderate acute hypercapnic respiratory failure, with an arterial pH < 7.30 and a PaCO_2_ ≥50 mmHg	AIRVO^TM^ 2, Fisher and Paykel Healthcare, Auckland, New Zealand	Philips V60	67.9 ± 6.9/72.3 ± 7.8	HFNC: the air temperature was set at 37°C at a flow rate of 30–55 L/min. For NIV, the inspiratory positive airway pressure (IPPV) was set at 10 cm H_2_O, and expiration pressure was set at 5 cm H_2_O	Both HFNC and NIV were used for three days after extubation	The primary endpoint was the rate of reintubation within 7 days of extubation. Secondary clinical endpoints included: Blood gases, complications, duration of endotracheal intubation mechanical ventilation, length of RICU stay, length of hospital stay, and mortality

We have no registration number for our study is not a clinical study but a meta-analysis.

## Data Availability

The data used to support this study are available within the article.
